# Decentralized Sliding Mode Observer Based Dual Closed-Loop Fault Tolerant Control for Reconfigurable Manipulator against Actuator Failure

**DOI:** 10.1371/journal.pone.0129315

**Published:** 2015-07-16

**Authors:** Bo Zhao, Chenghao Li, Derong Liu, Yuanchun Li

**Affiliations:** 1 Department of Control Engineering, Changchun University of Technology, Changchun, China; 2 State Key Laboratory of Management and Control for Complex Systems, Institute of Automation, Chinese Academy of Sciences, Beijing, China; 3 Product Development Department, FAW Car Co., Ltd, Changchun, China; University of California Berkeley, UNITED STATES

## Abstract

This paper considers a decentralized fault tolerant control (DFTC) scheme for reconfigurable manipulators. With the appearance of norm-bounded failure, a dual closed-loop trajectory tracking control algorithm is proposed on the basis of the Lyapunov stability theory. Characterized by the modularization property, the actuator failure is estimated by the proposed decentralized sliding mode observer (DSMO). Moreover, the actuator failure can be treated in view of the local joint information, so its control performance degradation is independent of other normal joints. In addition, the presented DFTC scheme is significantly simplified in terms of the structure of the controller due to its dual closed-loop architecture, and its feasibility is highly reflected in the control of reconfigurable manipulators. Finally, the effectiveness of the proposed DFTC scheme is demonstrated using simulations.

## Introduction

With the potential applications ranging from space exploration to smart manufacturing, from high risk tasks to battle fields, reconfigurable manipulators [1–4] that consist of interchangeable links and joint modules with standard connecting interfaces have been widely investigated for their modularization property. However, failures in actuators, sensors or mechanical components will inevitably occur after working for a long time in a variety of unknown or changeable environments that human can not intervene directly. A developing and untreated failure may lead the system to unstable stages that are dangerous to the entire workspace, or even harmful to human operators. Hence, fault tolerant control (FTC), which can provide higher safety and reliability, has become an urgent demand.

FTC is an active research field and many different methods have been proposed over the past several decades. FTC strategies can mainly be classified into two categories [5]: hardware redundancy based FTC [6–8] and analytical redundancy based FTC [10–12]. Hardware redundancy can be applied to solve a large class of FTC problems, but it requires some backup components which increase the weight, volume and cost. Compared to the hardware redundancy approach, analytical redundancy is distinguished for its low costs and flexible structures. Therefore, the analytical redundancy based FTC is increasingly applied due to its advantages. In terms of practical applications, the analytical redundancy based FTC consists of two specific approaches: passive FTC and active FTC. For both of them, the state estimation plays an important role, which can be grouped into three types. The first type is the least-squares estimation. For example, Hu et al. [9] established a recursive least square algorithm to identify and calibrate the model of lithium-ion battery online. He et al. [10] utilized the least-squares filter to minimize the estimation variance for the addressed time-varying networked sensing systems, and a novel residual matching approach was developed to isolate and estimate the fault. The second type is based on the filtering technique. Park et al. [11] have built a federated Kalman filter for fault detection and isolation (FDI) in order to improve the accuracy of the state estimation, and then a FTC scheme was proposed for actuator and sensor faults of a tilt-rotor unmanned aerial vehicle system. An *H*_∞_ filtering fault estimator for switched time-delay systems with impulsive control [12] was designed based on the assumed uniformly and differentiable bounded delay signal. And then a hybrid controller which was composed of a fault estimator and an impulsive controller was constructed. Sun et al. [13] presented an adaptive unscented Kalman filtering method based on covariance matching to estimate state of charge of a lithium-ion battery. Zhang et al. [14] utilized a extended Kalman filter (EKF) to recursively estimate the model parameters in the dynamic stress test on a specially established test rig, and then a model-based regulation and management for a reliable and safe operation was realized. Hu et al. [15] pointed out that the robust extended Kalman filter based estimation method possessed slightly smaller root-mean-square error, which leads to a better fault tolerant capability than the standard EKF. The third type is based on the observer approaches. A sliding mode observer based actuator fault accommodation method [16] was proposed to compensate the fault of near-space hypersonic vehicle. In [17], the estimated error obtained by reaction force observer was utilized for fault detection, and then exchanged the faulty sensor signal by the estimated one to maintain the fault-mode controller. By combining the adaptive backstepping technique with dynamic surface control approach and fuzzy state observer, an output-feedback fault tolerant control approach was developed in [18]. Based on the information from a standard bank of observers which match the different fault situations, an FDI module was able to reconfigure the control loop by selecting the appropriate control law from a bank of controllers [19]. By choosing a safe-park point, an active FTC [20] was developed through comparing the actuators’ output and the obtained fault information. Liu et al. [21] investigated an integral-type sliding mode FTC scheme against sensor failure for a class of nonlinear Itô stochastic systems by using an observer based state estimation. Apart from the above, some other FTC strategies with no need for estimating the state have been investigated as well. Shen et al. [22] considered the robust FTC for uncertain fractional-order systems by using reciprocal projection lemma and some properties of Kronecker product. Under the consideration of inertia uncertainties, external disturbances, actuator failures and saturations, a novel non-singular terminal sliding-mode control law [23] was designed for spacecraft systems to obtain finite-time convergence and high control precision. A sliding mode control based FTC for the attitude stabilization [24] was derived to avoid the partial loss of actuator effectiveness. In [25], a general active FTC framework was proposed for nonlinear systems with sensor failures. A fault detection and isolation module was first built for alarming the time and location of sensor fault occurrence. Moreover, a reconfiguration-based fault tolerance scheme [26] that mixed the passive and active approaches was designed to recover all the recoverable faults based on the concept of bottom-up extensible controls. For a flight control system with partial actuator failures, a hybrid FTC system, which combined the merits of passive and active FTC, was presented in [8] to counteract the faults through an optimal reconfigurable controller.

Besides, several literatures concerned FDI and FTC of reconfigurable manipulators. Inspired by the relationship between power efficiency degradation and operation health conditions, an effective power efficiency estimation-based health monitoring and fault detection technique [27] was developed for modular and reconfigurable robots (MRR) with a joint torque sensor. Power efficiency coefficients of each joint module were obtained using sensor measurements and used directly for health monitoring and fault detection. In [28], a distributed fault detection scheme was proposed by comparing the filtered joint torque command with the estimated one derived from the nonlinear dynamic model of MRR with joint torque sensing. In [29], a universal approach to configuration synthesis of reconfigurable robots was proposed based on fault tolerant indices. Liu et al. [30–31] proposed a DFTC based on an observer for fault detection of MRR with the joint torque sensing, which was independent of joint control approach. Zhao et al. [32] investigated an FDI scheme based on a sliding mode observer for reconfigurable manipulator with sensor fault. And for the actuator fault identification, an unknown input state observer [33] was exploited.

In our previous work [32], a sensor fault identification scheme was concerned, rather than a fault tolerant control. Different from it, in this study, the goal of the satisfactory control performance after the fault being detected and identified is achieved for reconfigurable manipulators with actuator failure. Motivated by [32] and the aforementioned literature, an observer and dual closed-loop based FTC protocol is developed to deal with the trajectory tracking problem with the consideration of possible actuator failure. Meanwhile, a DSMO is essentially adopted to generate the residual signal for the purpose of fault tolerance. With the occurrence of actuator failure, the dual closed-loop FTC can be actively reconfigured according to the residual information. Moreover, the weights of neural networks are promptly updated using the proposed adaptive laws.

The main contributions and merits of this work are as follows: (i) It is more feasible to develop a DFTC scheme for reconfigurable manipulators with their modularization property, especially when they consist of a plenty of modules. (ii) The real-time estimated actuator failure, which is obtained by the DSMO, is embedded in the controller design. Thus, it does not need to reconfigure the controller when the fault occurs. (iii) The failure can be handled in the faulty subsystem, rather than the entire system. Therefore, the control performance degradation of the faulty subsystem can not reflect the normal subsystems. (iv) Along with the dual closed-loop fault tolerant control architecture, the control structure is properly simplified, and the feasibility of the proposed controller is further enhanced.

This paper is organized as follows. The problem formulation briefly describes the mathematical model with/without actuator failure. Subsequently, a DFTC scheme is proposed to handle the actuator failure and the stability proof is given in detail based on Lyapunov stability theory. Finally, the numerical simulation results are shown, and a discussion and conclusion are presented.

## Methods

### Problem formulation

The dynamic model of *n*-DOF reconfigurable manipulator with joint dynamic friction can be described as

$$\begin{array}{c} M\left(q\right)\ddot{q}+C\left(q,\dot{q}\right)\dot{q}+G\left(q\right)+F\left(q,\dot{q}\right)=u \end{array}$$ (1)

where *q* ∈ *R* ^*n*^ is the vector of joint displacements, *M*(*q*) ∈ *R* ^*n* × *n*^ is the positive definite inertia matrix, $C(q,\dot{q})\dot{q}\in R^{n}$ is the Coriolis and centripetal force, *G*(*q*) ∈ *R* ^*n*^ is the gravity term, $F(q,\dot{q})\in R^{n}$ is the vector of joint friction torques, and *u* ∈ *R* ^*n*^ is the applied joint torque.

In engineering practice, such as space manipulating and disaster rescue, the reconfigurable manipulators usually consist of an uncertain or even large number of module joints, and it brings complex control structure and heavy computational burden. The traditional centralized control is difficult to apply. In order to release these limitations, each joint in the reconfigurable manipulator system is considered as a subsystem of the entire manipulator system interconnected by coupling torque. By separating terms only depending on local variables $\left(q_{i},{\dot{q}}_{i},{\overset{‥}{q}}_{i}\right)$ from those terms of other joint variables, each subsystem dynamical model can be formulated in joint space as [32]

$$\begin{array}{c} M_{i}\left(q_{i}\right){\ddot{q}}_{i}+C_{i}\left(q_{i},{\dot{q}}_{i}\right){\dot{q}}_{i}+G_{i}\left(q_{i}\right)\;+F_{i}\left(q_{i},{\dot{q}}_{i}\right)+Z_{i}\left(q,\dot{q},\ddot{q}\right)=u_{i} \end{array}$$ (2)

$$\begin{array}{ccc} Z_{i}\left(q,\dot{q},\ddot{q}\right) & = & \{\sum_{j=1,j\neq i}^{n}M_{ij}\left(q\right){\ddot{q}}_{j}+\left[M_{ii}\left(q\right)-M_{i}\left(q_{i}\right)\right]{\ddot{q}}_{i}\}+\\ & & \{\sum_{j=1,j\neq i}^{n}C_{ij}\left(q,\dot{q}\right){\dot{q}}_{j}+\left[C_{ii}\left(q,\dot{q}\right)-C_{i}\left(q_{i},{\dot{q}}_{i}\right)\right]{\dot{q}}_{i}\}+\left[{\bar{G}}_{i}\left(q\right)-G_{i}\left(q_{i}\right)\right] \end{array}$$

where *q*_*i*_, ${\dot{q}}_{i}$, ${\overset{‥}{q}}_{i}$, *G*_*i*_(*q*), $F_{i}(q_{i},{\dot{q}}_{i})$ and *u*_*i*_ are the *i-*th element of the vectors *q*, $\dot{q}$, $\overset{‥}{q}$, *G*(*q*), $F(q,\dot{q})$ and *u*, respectively. *M*_*ij*_(*q*) and $C_{ij}(q,\dot{q})$ are the *ij-*th element of the matrices *M*(*q*) and $C(q,\dot{q})$, respectively.

By taking the actuator failure into account, the subsystem dynamic model can be expressed as

$$\begin{array}{c} M_{i}\left(q_{i}\right){\ddot{q}}_{i}+C_{i}\left(q_{i},{\dot{q}}_{i}\right){\dot{q}}_{i}+G_{i}\left(q_{i}\right)+F_{i}\left(q_{i},{\dot{q}}_{i}\right)+Z_{i}\left(q,\dot{q},\ddot{q}\right)+f_{ia}\left(q_{i},{\dot{q}}_{i}\right)=u_{i} \end{array}$$ (3)

where $f_{ia}(q_{i},{\dot{q}}_{i})=\alpha (t-T_{ia})\psi_{i}(q_{i},{\dot{q}}_{i})$ is the term for actuator failure, *α*(*t* − *T*_*ia*_) is the step function, *T*_*ia*_ is the time when the actuator failure occurs in the *i-*th subsystem, and $\psi_{i}(q_{i},{\dot{q}}_{i})$ is the actuator failure function.

Let *x*_*i*1_ = *q*_*i*_. Then Eq (3) should be described as

$$\begin{array}{c} \left\{ \begin{array}{c} {\dot{x}}_{i1}=x_{i2}\\ {\dot{x}}_{i2}=f_{i}\left(q_{i},{\dot{q}}_{i}\right)+g_{i}\left(q_{i}\right)\left[u_{i}+f_{ia}\left(q_{i},{\dot{q}}_{i}\right)\right]+h_{i}\left(q,\dot{q},\ddot{q}\right) \end{array}\right. \end{array}$$ (4)

where

$$\begin{array}{ccc} f_{i}\left(q_{i},{\dot{q}}_{i}\right) & = & M_{i}^{-1}\left(q_{i}\right)[-C_{i}\left(q_{i},{\dot{q}}_{i}\right){\dot{q}}_{i}-G_{i}\left(q_{i}\right)-F_{i}\left(q_{i},{\dot{q}}_{i}\right)]\\ g_{i}\left(q_{i}\right) & = & M_{i}^{-1}\left(q_{i}\right)\\ h_{i}\left(q,\dot{q},\ddot{q}\right) & = & -M_{i}^{-1}\left(q_{i}\right)Z_{i}\left(q,\dot{q},\ddot{q}\right). \end{array}$$ (5)

**Assumption 1** [34]: The time-varying actuator failure $f_{ia}(q_{i},{\dot{q}}_{i})$ is assumed to be an unknown signal under polynomial form of (*k* − 1)st degree, whose *k*-th time derivative is norm bounded by a positive scalar *δ*_*i*_. The following notations are used:

$$\begin{array}{c} \left\{ \begin{array}{c} {\dot{f}}_{ia}\left(q_{i},{\dot{q}}_{i}\right)=f_{ia1}\left(q_{i},{\dot{q}}_{i}\right)\\ {\dot{f}}_{ia1}\left(q_{i},{\dot{q}}_{i}\right)=f_{ia2}\left(q_{i},{\dot{q}}_{i}\right)\\ \cdots \\ ∥f_{iak}\left(q_{i},{\dot{q}}_{i}\right)∥\leq \delta_{i}. \end{array}\right. \end{array}$$ (6)

The main objective of this work is to develop a DFTC for (4), by which the trajectory tracking mission can be achieved with the occurrence of actuator failure. Furthermore, the simplicity of the proposed FTC is expected regarding the basic architecture of the reconfigurable manipulator. Moreover, the robustness of the control algorithm is supposed to be considered due to a variety of system parameters.

### Decentralized fault tolerant controller design

**Fig 1:**
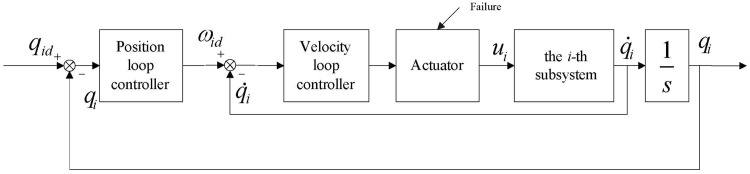
Control system architecture of *i-*th subsystem. The control architecture contains two closed-loops: position loop (outer loop) and velocity loop (inner loop).

Fig 1 shows the control system architecture of the *i-*th subsystem of the designed decentralized fault tolerant controller, which can make each joint’s output tracks its desired trajectory. The control architecture contains two closed-loops. In the outer position loop, the position tracking error and virtue velocity are taken as the input and output, respectively. In the inner velocity loop, the virtue velocity tracking error and the control torque are taken as the input and output, respectively. The detailed design procedure of dual closed-loop integral sliding mode based fault tolerant controller is described as follows.

#### Actuator failure estimation based on decentralized observer

A real-time estimation strategy is developed in this subsection, and the estimated information will be further adopted by the dual closed-loop DFTC scheme.

**Assumption 2**: The desired trajectories *q*_*id*_ are twice differentiable and bounded as

$$\begin{array}{c} ∥\begin{array}{c} q_{id}\\ {\dot{q}}_{id}\\ {\ddot{q}}_{id} \end{array}∥\leq q_{iA} \end{array}$$ (7)

where *q*_*iA*_ is a known positive scalar.

Design a decentralized neural network sliding mode observer (DNNSMO) as

$$\begin{array}{c} \left\{ \begin{array}{c} {\dot{\hat{x}}}_{i1}={\hat{x}}_{i2}+\upsilon_{i1}\\ {\dot{\hat{x}}}_{i2}={\hat{f}}_{io}\left({\hat{q}}_{i},{\dot{\hat{q}}}_{i},{\hat{\theta }}_{ifo}\right)+{\hat{g}}_{io}\left({\hat{q}}_{i},{\hat{\theta }}_{igo}\right)u_{i}+\nu_{i}\left(e_{i2},{\hat{\theta }}_{ipo}\right)+\upsilon_{i2}+{\hat{\delta }}_{i} \end{array}\right. \end{array}$$ (8)

where ${\hat{f}}_{io}$ and ${\hat{g}}_{io}$ are estimations of the uncertainty terms ${\hat{f}}_{i}$ and ${\hat{g}}_{i}$, respectively.

$$\begin{array}{c} \upsilon_{ij}=\kappa_{ij}\frac{x_{ij}-{\hat{x}}_{ij}}{∥x_{ij}-{\hat{x}}_{ij}∥}, \end{array}$$ (9)

*j* = 1,2 and ${\hat{\delta }}_{i}$ is used to compensate the observed error.

Define the observed error $e_{i1}=x_{i1}-{\hat{x}}_{i1}$ and $e_{i2}=x_{i2}-{\hat{x}}_{i2}$. From Eqs (4) and (8), the error dynamic model can be expressed as

$$\begin{array}{c} \left\{ \begin{array}{c} {\dot{e}}_{i1}=e_{i2}-\upsilon_{i1}\\ {\dot{e}}_{i2}=\left(f_{i}-{\hat{f}}_{io}\right)+\left(g_{i}-{\hat{g}}_{io}\right)u_{i}+h_{i}-\nu_{i}+g_{io}f_{ia}-\upsilon_{i2}-{\hat{\delta }}_{i}. \end{array}\right. \end{array}$$ (10)

The following ideal radial basis function (RBF) neural networks are used to approximate the unknown terms $f_{i}(q_{i},{\dot{q}}_{i})$ and *g*_*i*_(*q*_*i*_), respectively,

$$\begin{array}{c} f_{i}\left(q_{i},{\dot{q}}_{i}\right)=\theta_{if}^{T}\sigma_{if}\left(q_{i},{\dot{q}}_{i}\right)+\varepsilon_{if} \end{array}$$ (11)

$$\begin{array}{c} g_{i}\left(q_{i}\right)=\theta_{ig}^{T}\sigma_{ig}\left(q_{i}\right)+\varepsilon_{ig}. \end{array}$$ (12)

Then, they are observed by ${\hat{f}}_{io}({\hat{q}}_{i},{\dot{\hat{q}}}_{i},{\hat{\theta }}_{ifo})$ and ${\hat{g}}_{io}({\hat{q}}_{i},{\hat{\theta }}_{igo})$ as

$$\begin{array}{c} {\hat{f}}_{io}\left({\hat{q}}_{i},{\dot{\hat{q}}}_{i},{\hat{\theta }}_{ifo}\right)={\hat{\theta }}_{ifo}^{T}{\hat{\sigma }}_{ifo}\left({\hat{q}}_{i},{\dot{\hat{q}}}_{i}\right) \end{array}$$ (13)

$$\begin{array}{c} {\hat{g}}_{io}\left({\hat{q}}_{i},{\hat{\theta }}_{igo}\right)={\hat{\theta }}_{igo}^{T}{\hat{\sigma }}_{igo}\left({\hat{q}}_{i}\right) \end{array}$$ (14)

where ${\hat{\theta }}_{ifo}$ and ${\hat{\theta }}_{igo}$ are the estimations of the ideal neural network weights *θ*_*if*_ and *θ*_*ig*_, respectively. *ɛ*_*if*_ and *ɛ*_*ig*_ are the residual errors, and ${\tilde{\theta }}_{ifo}={\hat{\theta }}_{ifo}-\theta_{if}$ and ${\tilde{\theta }}_{igo}={\hat{\theta }}_{igo}-\theta_{ig}$ are the estimated errors of the weight vectors.

**Assumption 3**: The interconnection term $h_{i}(q,\dot{q},\overset{‥}{q})$ is bounded as [35]

$$\begin{array}{c} |h_{i}\left(q,\dot{q},\ddot{q}\right)|\leq \sum_{j=1}^{n}d_{ij}E_{j}\leq n\;\max_{ij}\{d_{ij}\}E_{i} \end{array}$$ (15)

where *d*_*ij*_ ≥ 0 and *E*_*j*_ = 1+∣*e*_*j*2_∣+∣*e*_*j*2_∣^2^.

Define $R_{io}(e_{i2},\theta_{ip})=n\;\max_{ij}\left\{d_{ij}\right\}E_{i}$, which is approximated by the following ideal RBF neural network,

$$\begin{array}{c} R_{io}\left(e_{i2},\theta_{ip}\right)=\theta_{ip}\sigma_{ip}\left(|e_{i2}|\right)+\varepsilon_{ip} \end{array}$$ (16)

Its observation is

$$\begin{array}{c} \nu_{i}\left(e_{i2},{\hat{\theta }}_{ipo}\right)=\text{sgn}\left(e_{i2}\right){\hat{\theta }}_{ipo}{\hat{\sigma }}_{ipo}\left(|e_{i2}|\right) \end{array}$$ (17)

where ${\hat{\theta }}_{ipo}$ is the estimation of the weight *θ*_*ip*_, ${\hat{\sigma }}_{ipo}$ is the estimation of neural network basis function *σ*_*ip*_, ${\tilde{\theta }}_{ipo}={\hat{\theta }}_{ipo}-\theta_{ip}$ and ${\tilde{\sigma }}_{ipo}={\hat{\sigma }}_{ipo}-\sigma_{ip}$ are defined as their estimated errors.

The estimated errors are defined as

$$\begin{array}{ccc} \omega_{i1} & = & \theta_{if}^{T}({\hat{\sigma }}_{ifo}\left({\hat{q}}_{i}\text{,}{\dot{\hat{q}}}_{i}\right)-\sigma_{if}\left(q_{i}\text{,}{\dot{q}}_{i}\right))+\varepsilon_{if}+\varepsilon_{ig}u_{i}\\ & & +\;\theta_{ig}^{T}\left({\hat{\sigma }}_{igo}\left({\hat{q}}_{i}\right)-\sigma_{ig}\left(q_{i}\right)\right)u_{i}+\left(g_{io}f_{ia}-{\hat{g}}_{io}\hat{f_{ia}}\right) \end{array}$$ (18)

$$\begin{array}{c} \omega_{i2}=R_{i}\left(e_{i2},\theta_{ip}\right)-\theta_{ip}^{T}{\hat{\sigma }}_{ipo}\left(|e_{i2}|\right). \end{array}$$ (19)

They are unknown but bounded, we define

$$\begin{array}{c} \omega_{i}=|\omega_{i1}|+|\omega_{i2}|. \end{array}$$ (20)

The RBF neural network weights ${\hat{\theta }}_{ifo},{\hat{\theta }}_{igo},{\hat{\theta }}_{ipo}$ and ${\hat{\delta }}_{i}$ are updated by the following adaptation laws

$$\begin{array}{c} {\dot{\hat{\theta }}}_{ifo}=-\eta_{ifo}e_{i2}{\hat{\sigma }}_{ifo} \end{array}$$ (21)

$$\begin{array}{c} {\dot{\hat{\theta }}}_{igo}=-\eta_{igo}e_{i2}{\hat{\sigma }}_{igo}\left(q_{i}\right)u_{i} \end{array}$$ (22)

$$\begin{array}{c} {\dot{\hat{\theta }}}_{ipo}=-\eta_{ipo}|e_{i2}|{\hat{\sigma }}_{ipo} \end{array}$$ (23)

$$\begin{array}{c} {\dot{\hat{\delta }}}_{i}=\lambda_{i}e_{i2} \end{array}$$ (24)

where *η*_*ifo*_, *η*_*igo*_, *η*_*ipo*_ and *λ*_*i*_ are positive constants.

**Theorem 1**: Consider the error dynamics Eq (10), and combine the assumptions 2 and 3 and the adaptation laws Eqs (21)–(24). The actuator failure can be estimated by the designed DSMO Eq (8) in real-time, which implies that the observed error *e*_*i*_ converges to zero asymptotically, i.e., the estimated state ${\hat{x}}_{i}$ tracks the actual state *x*_*i*_.

Considering the description of Eq (9), select a proper positive constant *σ*_*i*_, the equivalent output error can be estimated as follows with any precision if *κ*_*i*2_ > ‖*f*_*ia*_‖ holds [32],

$$\begin{array}{c} \upsilon_{i2\sigma }=\kappa_{i2}\frac{x_{i2}-{\hat{x}}_{i2}}{∥x_{i2}-{\hat{x}}_{i2}∥+\sigma_{i}}. \end{array}$$ (25)

The actuator failure can be obtained by

$$\begin{array}{c} {\hat{f}}_{ia}={\hat{g}}_{io}^{-1}\upsilon_{i2\sigma }. \end{array}$$ (26)

**Proof**: Select the Lyapunov function candidate as

$$\begin{array}{c} V_{i}=V_{i1}+V_{i2}. \end{array}$$ (27)

**Step 1**: Select a sub-Lyapunov function as

$$\begin{array}{c} V_{i1}=\frac{1}{2}e_{i1}^{2}. \end{array}$$ (28)

Its time derivative is

$$\begin{array}{c} {\dot{V}}_{i1}=e_{i1}{\dot{e}}_{i1}\leq |e_{i1}|\left(|e_{i2}|-\kappa_{i1}\right). \end{array}$$ (29)

Thus ${\dot{V}}_{1}=\sum_{i=1}^{n}\dot{V_{i1}}\leq \sum_{i=1}^{n}\left(-\mu_{i1}|e_{i1}|\right)<0$ if *κ*_*i*1_ > *μ*_*i*1_+∣*e*_*i*2_∣ holds, where *μ*_*i*1_ is a small positive constant.

**Step 2**: Let ${\tilde{f}}_{ia}=f_{ia}-{\hat{f}}_{ia}$, select another sub-Lyapunov function as

$$\begin{array}{c} V_{i2}=\frac{1}{2}e_{i2}^{2}+\frac{1}{2\eta_{ifo}}{\tilde{\theta }}_{ifo}^{T}{\tilde{\theta }}_{ifo}+\frac{1}{2\eta_{igo}}{\tilde{\theta }}_{igo}^{T}{\tilde{\theta }}_{igo}+\frac{1}{2\eta_{ipo}}{\tilde{\theta }}_{ipo}^{2}+\frac{1}{2\lambda_{i}}{\tilde{\delta }}_{i}^{2}. \end{array}$$ (30)

Considering Eqs (10), (18), (21), (22), (26) and assumption 3, the time derivative of Eq (30) is

$$\begin{array}{ccc} {\dot{V}}_{i2} & = & e_{i2}[\left(f_{i}-{\hat{f}}_{io}\right)+\left(g_{i}-{\hat{g}}_{io}\right)u_{i}+h_{i}-\nu_{i}+g_{i}f_{ia}\\ & & -\upsilon_{i2\sigma }-{\hat{\delta }}_{i}]+\frac{1}{\eta_{ifo}}{\tilde{\theta }}_{ifo}^{T}{\dot{\hat{\theta }}}_{ifo}+\frac{1}{\eta_{igo}}{\tilde{\theta }}_{igo}^{T}{\dot{\hat{\theta }}}_{igo}+\frac{1}{\eta_{ipo}}{\tilde{\theta }}_{ipo}{\dot{\hat{\theta }}}_{ipo}-\frac{1}{\lambda_{i}}{\tilde{\delta }}_{i}{\dot{\hat{\delta }}}_{i}\\ & \leq & {\tilde{\theta }}_{ifo}^{T}\left(e_{i2}{\hat{\sigma }}_{ifo}+\frac{1}{\eta_{ifo}}{\dot{\hat{\theta }}}_{ifo}\right)+{\tilde{\theta }}_{igo}^{T}\left(e_{i2}{\hat{\sigma }}_{igo}u_{i}+\frac{1}{\eta_{igo}}{\dot{\hat{\theta }}}_{igo}\right)\\ & & +|e_{i2}||\omega_{i1}|+e_{i2}h_{i}-|e_{i2}|{\hat{\theta }}_{ipo}{\hat{\sigma }}_{ipo}-e_{i2}{\hat{\delta }}_{i}+\frac{1}{\eta_{ipo}}{\tilde{\theta }}_{ipo}{\dot{\hat{\theta }}}_{ipo}-\frac{1}{\lambda_{i}}{\tilde{\delta }}_{i}{\dot{\hat{\delta }}}_{i}\\ & \leq & -|e_{i2}|{\hat{\theta }}_{ipo}{\hat{\sigma }}_{ipo}+|e_{i2}||\omega_{i1}|-e_{i2}{\hat{\delta }}_{i}+\frac{1}{\eta_{ipo}}{\tilde{\theta }}_{ipo}{\dot{\hat{\theta }}}_{ipo}\\ & & -\frac{1}{\lambda_{i}}{\tilde{\delta }}_{i}{\dot{\hat{\delta }}}_{i}+|e_{i2}|n\;\max_{ij}\{d_{ij}\}E_{i} \end{array}$$ (31)

Defining ${\tilde{\delta }}_{i}=\omega_{i}-{\hat{\delta }}_{i}$, and substituting Eqs (19), (23), (24) into Eq (31) yields

$$\begin{array}{ccc} {\dot{V}}_{2} & \leq & \sum_{i=1}^{n}(|e_{i2}|\left(R_{i}\left(|e_{i2}|\right)-{\hat{\theta }}_{ipo}{\hat{\sigma }}_{ipo}\right)+|e_{i2}||\omega_{i1}|-e_{i2}{\hat{\delta }}_{i}+\frac{1}{\eta_{ipo}}{\tilde{\theta }}_{ipo}{\dot{\hat{\theta }}}_{ipo}-\frac{1}{\lambda_{i}}{\tilde{\delta }}_{i}{\hat{\delta }}_{i})\\ & \leq & \sum_{i=1}^{n}(\left(|e_{i2}|{\tilde{\theta }}_{ipo}{\hat{\sigma }}_{ipo}+\frac{1}{\eta_{ipo}}{\tilde{\theta }}_{ipo}{\dot{\hat{\theta }}}_{ipo}\right)+|e_{i2}||\omega_{i1}|+|e_{i2}||\omega_{i2}|-e_{i2}{\hat{\delta }}_{i}-\frac{1}{\lambda_{i}}{\tilde{\delta }}_{i}{\hat{\delta }}_{i})\\ & = & \sum_{i=1}^{n}(|e_{i2}|\left(\omega_{i}-{\hat{\delta }}_{i}\right)-\frac{1}{\lambda_{i}}{\tilde{\delta }}_{i}{\hat{\delta }}_{i})=0. \end{array}$$ (32)

From the previous two steps, the time derivative of Eq (27) is obtained as

$$\begin{array}{c} \dot{V}=\sum_{i=1}^{n}\left({\dot{V}}_{i1}+{\dot{V}}_{i2}\right)<0. \end{array}$$ (33)

According to Lyapunov stability theory and Barbalat Lemma, the state observed error *e*_*i*_ will converge to zero asymptotically.

#### Decentralized dual closed-loop fault tolerant controller design

Define the position loop tracking error as

$$\begin{array}{c} e_{ip}=q_{id}-q_{i}. \end{array}$$ (34)

The position loop integral sliding mode surface is defined as

$$\begin{array}{c} s_{ip}=e_{ip}+k_{i1}\int_{0}^{t}e_{ip}d\tau \end{array}$$ (35)

where *k*_*i*1_ > 0. Its time derivative is

$$\begin{array}{c} {\dot{s}}_{ip}={\dot{e}}_{ip}+k_{i1}e_{ip}={\dot{q}}_{id}-{\dot{q}}_{i}+k_{i1}e_{ip}. \end{array}$$ (36)

Define the velocity loop tracking error as

$$\begin{array}{c} e_{iv}=\omega_{id}-{\dot{q}}_{i} \end{array}$$ (37)

where *ω*_*id*_ is the position-loop control law to be designed (Fig 1).

Substituting Eq (37) into Eq (36) to get

$$\begin{array}{c} {\dot{s}}_{ip}={\dot{e}}_{ip}+k_{i1}e_{ip}={\dot{q}}_{id}-\omega_{id}+e_{iv}+k_{i1}e_{ip}. \end{array}$$ (38)

Design the position-loop control law as

$$\begin{array}{c} \omega_{id}={\dot{q}}_{id}+k_{i1}e_{ip}+\rho_{i1}s_{ip}+e_{iv} \end{array}$$ (39)

where *ρ*_*i*1_ > 0.

The velocity loop integral sliding mode surface is given as

$$\begin{array}{c} s_{iv}=e_{iv}+k_{i2}\int_{0}^{t}e_{iv}d\tau \end{array}$$ (40)

where *k*_*i*2_ > 0. Its time derivative is

$$\begin{array}{ccc} {\dot{s}}_{iv} & = & {\dot{e}}_{iv}+k_{i2}e_{iv}={\dot{\omega }}_{id}-{\ddot{q}}_{i}+k_{i2}e_{iv}\\ & = & {\dot{\omega }}_{id}-f_{i}\left(q_{i},{\dot{q}}_{i}\right)-g_{i}\left(q_{i}\right)\left(u_{i}+f_{ia}\right)-h_{i}\left(q,\dot{q},\ddot{q}\right)+k_{i2}e_{iv}. \end{array}$$ (41)

Considering Eqs (11) and (12), $f_{i}(q_{i},{\dot{q}}_{i})$ and *g*_*i*_(*q*_*i*_) should be estimated by ${\hat{f}}_{i}(q_{i},{\dot{q}}_{i})$ and ${\hat{g}}_{i}(q_{i})$ as

$$\begin{array}{c} {\hat{f}}_{i}\left(q_{i},{\dot{q}}_{i}\right)={\hat{\theta }}_{if}^{T}{\hat{\sigma }}_{if}\left(q_{i},{\dot{q}}_{i}\right) \end{array}$$ (42)

$$\begin{array}{c} {\hat{g}}_{i}\left(q_{i}\right)={\hat{\theta }}_{ig}^{T}{\hat{\sigma }}_{ig}\left(q_{i}\right) \end{array}$$ (43)

where ${\hat{\sigma }}_{if}$ and ${\hat{\sigma }}_{ig}$ are the estimations of *σ*_*if*_ and *σ*_*ig*_, and ${\hat{\theta }}_{if}$ and ${\hat{\theta }}_{ig}$ are the estimations of *θ*_*if*_ and *θ*_*ig*_, respectively. The estimated errors are

$$\begin{array}{c} {\tilde{f}}_{i}={\hat{f}}_{i}(q_{i}\text{,}{\dot{q}}_{i})-f_{i}(q_{i}\text{,}{\dot{q}}_{i})={\tilde{\theta }}_{if}^{T}{\hat{\sigma }}_{if}(q_{i}\text{,}{\dot{q}}_{i})+\theta_{if}^{T}{\tilde{\sigma }}_{if}(q_{i}\text{,}{\dot{q}}_{i})-\varepsilon_{if} \end{array}$$ (44)

$$\begin{array}{c} {\tilde{g}}_{i}={\tilde{g}}_{i}\left(q_{i}\right)-g_{i}\left(q_{i}\right)={\tilde{\theta }}_{ig}^{T}{\hat{\sigma }}_{ig}\left(q_{i}\right)+\theta_{ig}^{T}{\tilde{\sigma }}_{ig}\left(q_{i}\right)-\varepsilon_{ig} \end{array}$$ (45)

where ${\tilde{\theta }}_{if}={\hat{\theta }}_{if}-\theta_{if}$ and ${\tilde{\theta }}_{ig}={\hat{\theta }}_{ig}-\theta_{ig}$.

**Assumption 4**: The interconnection term $h_{i}(q,\dot{q},\overset{‥}{q})$ is bounded by

$$\begin{array}{c} |h_{i}\left(q,\dot{q},\ddot{q}\right)|\leq \sum_{j=1}^{n}s_{ij}S_{j}\leq n\;\max_{ij}\{s_{ij}\}S_{i} \end{array}$$ (46)

where *s*_*ij*_ ≥ 0 is an unknown constant, and *S*_*j*_ = 1+∣*s*_*jv*_∣+∣*s*_*jv*_∣^2^.

Define $R_{i}(s_{iv},\theta_{ip}^{\prime })=n\;\max_{ij}\left\{s_{ij}\right\}S_{i}$, and the following ideal RBF neural network is employed to approximate it

$$\begin{array}{c} R_{i}\left(s_{iv},\theta_{ip}^{{}^{\prime }}\right)=\theta_{ip}^{{}^{\prime }}\sigma_{ip}\left(|s_{iv}|\right)+\varepsilon_{ip}^{{}^{\prime }}. \end{array}$$ (47)

The velocity loop controller should be designed as

$$\begin{array}{ccc} u_{i} & = & {\hat{g}}_{i}^{-1}\left(q_{i},{\hat{\theta }}_{ig}\right)[-{\hat{f}}_{i}\left(q_{i},{\dot{q}}_{i},{\hat{\theta }}_{if}\right)-v_{i}\left(s_{iv},{\hat{\theta }}_{ip}\right)+{\dot{\omega }}_{id}+k_{i2}e_{iv}\\ & & +\mu_{i}s_{iv}+\rho_{i2}\text{sgn}\left(s_{iv}\right)]-{\hat{f}}_{ia}\left(q_{i},{\dot{q}}_{i}\right) \end{array}$$ (48)

where $v_{i}(s_{iv},{\hat{\theta }}_{ip})$ is adopted to compensate the interconnection term, and it can be expressed as

$$\begin{array}{c} v_{i}\left(s_{iv},{\hat{\theta }}_{ip}\right)=-\text{sgn}\left(s_{iv}\right){\hat{\theta }}_{ip}{\hat{\sigma }}_{ip}\left(|s_{iv}|\right) \end{array}$$ (49)

where ${\hat{\theta }}_{ip}{\hat{\sigma }}_{ip}(|s_{iv}|)$ is a neural network, ${\hat{\theta }}_{ip}$ is the estimation of the weight *θ*_*ip*_, the estimated error is defined as ${\tilde{\theta }}_{ip}={\hat{\theta }}_{ip}-\theta_{ip}$, ${\hat{\sigma }}_{ip}$ is the estimated value of *σ*_*ip*_, and the estimated error of Gaussian basis function is ${\tilde{\sigma }}_{ip}={\hat{\sigma }}_{ip}-\sigma_{ip}$.

Define the approximated error of neural networks as

$$\begin{array}{c} w_{i1}=\theta_{if}^{T}{\tilde{\sigma }}_{if}(q_{i}\text{,}{\dot{q}}_{i})-\varepsilon_{if}+\theta_{ig}^{T}{\tilde{\sigma }}_{ig}\left(q_{i}\right)u_{i}-\varepsilon_{ig}u_{i}+g_{i}(f_{ia}-{\hat{f}}_{ia}) \end{array}$$ (50)

$$\begin{array}{c} w_{i2}=R_{i}\left(s_{iv},\theta_{ip}^{{}^{\prime }}\right)-\theta_{ip}^{T}{\hat{\sigma }}_{ip}(|s_{iv}|) \end{array}$$ (51)

$$\begin{array}{c} w_{i}=|w_{i1}|+|w_{i2}|. \end{array}$$ (52)

The weights ${\hat{\theta }}_{if}$, ${\hat{\theta }}_{ig}$ and ${\hat{\theta }}_{ip}$ of the RBF neural networks are updated by the following adaptation laws

$$\begin{array}{c} {\dot{\hat{\theta }}}_{if}=-\eta_{if}s_{iv}{\hat{\sigma }}_{if} \end{array}$$ (53)

$$\begin{array}{c} {\dot{\hat{\theta }}}_{ig}=-\eta_{ig}s_{iv}{\hat{\sigma }}_{ig}u_{i} \end{array}$$ (54)

$$\begin{array}{c} {\dot{\hat{\theta }}}_{ip}=-\eta_{ip}|s_{iv}|{\hat{\sigma }}_{ip}. \end{array}$$ (55)

**Theorem 2**: Consider the subsystem dynamic model with actuator failure Eq (4), by virtue of the decentralized controller Eqs (39) and (48) with the parameter adaptation laws Eqs (53)–(55), the state tracking error will converge to zero asymptotically. In other words, the DFTC is achieved.

**Proof**: Considering the following Lyapunov function candidate

$$\begin{array}{c} V=V_{1}+V_{2} \end{array}$$ (56)

where

$$\begin{array}{c} V_{1}=\sum_{i=1}^{n}\frac{1}{2}s_{ip}^{2} \end{array}$$ (57)

$$\begin{array}{c} V_{2}=\sum_{i=1}^{n}(\frac{1}{2}s_{iv}^{2}+\frac{1}{2\eta_{if}}{\tilde{\theta }}_{if}^{T}{\tilde{\theta }}_{if}+\frac{1}{2\eta_{ig}}{\tilde{\theta }}_{ig}^{T}{\tilde{\theta }}_{ig}+\frac{1}{2\eta_{ip}}{\tilde{\theta }}_{ip}^{2}). \end{array}$$ (58)

Step1: The time derivative of *V*_1_ is

$$\begin{array}{ccc} {\dot{V}}_{1} & = & \sum_{i=1}^{n}s_{ip}{\dot{s}}_{ip}\\ & = & -\sum_{i=1}^{n}\rho_{i1}s_{ip}^{2}\leq 0. \end{array}$$ (59)

Step 2: The time derivative of *V*_2_ is

$$\begin{array}{c} {\dot{V}}_{2}=\sum_{i=1}^{n}(s_{iv}{\dot{s}}_{iv}+\frac{1}{\eta_{if}}{\tilde{\theta }}_{if}^{T}{\dot{\hat{\theta }}}_{if}+\frac{1}{\eta_{ig}}{\tilde{\theta }}_{ig}^{T}{\dot{\hat{\theta }}}_{ig}+\frac{1}{\eta_{ip}}{\tilde{\theta }}_{ip}{\dot{\hat{\theta }}}_{ip}). \end{array}$$ (60)

Substituting Eq (48) into Eq (60), one obtains

$$\begin{array}{ccc} {\dot{V}}_{2} & = & \sum_{i=1}^{n}[s_{iv}({\hat{f}}_{i}-f_{i}+\left({\hat{g}}_{i}-g_{i}\right)u_{i}-h_{i}\left(q,\dot{q},\ddot{q}\right)-v_{i}+g_{i}\left({\hat{f}}_{ia}-f_{ia}\right)\\ & & -\mu_{i}s_{iv}-\rho_{i2}\text{sgn}\left(s_{iv}\right))+\frac{1}{\eta_{if}}{\tilde{\theta }}_{if}{\dot{\hat{\theta }}}_{if}+\frac{1}{\eta_{ig}}{\tilde{\theta }}_{ig}{\dot{\hat{\theta }}}_{ig}+\frac{1}{\eta_{ip}}{\tilde{\theta }}_{ip}{\dot{\hat{\theta }}}_{ip}]\\ & \leq & \sum_{i=1}^{n}[{\tilde{\theta }}_{if}\left(s_{iv}{\hat{\sigma }}_{if}+\frac{1}{\eta_{if}}{\dot{\hat{\theta }}}_{if}\right)+{\tilde{\theta }}_{ig}\left(s_{iv}{\hat{\sigma }}_{ig}u_{i}+\frac{1}{\eta_{ig}}{\dot{\hat{\theta }}}_{ig}\right)+|s_{iv}||h_{i}|\\ & & +\;s_{iv}\omega_{i1}-\mu_{i}s_{iv}^{2}-\rho_{i2}|s_{iv}|-|s_{iv}|{\hat{\theta }}_{ip}{\hat{\sigma }}_{ip}+\frac{1}{\eta_{ip}}{\tilde{\theta }}_{ip}{\dot{\hat{\theta }}}_{ip}] \end{array}$$ (61)

Considering assumption 4, and substituting Eqs (52)–(55) into Eq (61), it follows that

$$\begin{array}{ccc} {\dot{V}}_{2} & \leq & \sum_{i=1}^{n}[|s_{iv}||w_{i1}|+|s_{iv}|\sum_{j=1}^{n}s_{ij}S_{j}-\mu_{i}s_{iv}^{2}-\rho_{i2}|s_{iv}|-|s_{iv}|{\hat{\theta }}_{ip}{\hat{\sigma }}_{ip}+\frac{1}{\eta_{ip}}{\tilde{\theta }}_{ip}{\dot{\hat{\theta }}}_{ip}]\\ & \leq & \sum_{i=1}^{n}[|s_{iv}||w_{i1}|-\mu_{i}s_{iv}^{2}-\rho_{i2}|s_{iv}|-|s_{iv}|{\hat{\theta }}_{ip}{\hat{\sigma }}_{ip}+\left(\underset{ij}{n\;\max\{s_{ij}\}}|s_{iv}|S_{i}+\frac{1}{\eta_{ip}}{\tilde{\theta }}_{ip}{\dot{\hat{\theta }}}_{ip}\right)]\\ & \leq & \sum_{i=1}^{n}[|s_{iv}||w_{i1}|-\mu_{i}s_{iv}^{2}-\rho_{i2}|s_{iv}|+\left(|s_{iv}|{\tilde{\theta }}_{ip}{\hat{\sigma }}_{ip}+\frac{1}{\eta_{ip}}{\tilde{\theta }}_{ip}{\dot{\hat{\theta }}}_{ip}\right)]\\ & \leq & \sum_{i=1}^{n}[|s_{iv}||w_{i1}|+|s_{iv}||w_{i2}|-\mu_{i}s_{iv}^{2}-\rho_{i2}|s_{iv}|]\\ & \leq & \sum_{i=1}^{n}[-\mu_{i}s_{iv}^{2}-\left(\rho_{i2}-w_{i}\right)|s_{iv}|]. \end{array}$$ (62)

It is clear that ${\dot{V}}_{2}\leq 0$ if the selected parameter *ρ*_*i*2_ > *w*_*i*_.

Note that $\dot{V}={\dot{V}}_{1}+{\dot{V}}_{2}\leq 0$ holds if the parameters selected as indicated above. Based on the Lyapunov stability theory and Barbalat Lemma, the system state error $e_{i}={\left[\begin{array}{cc} e_{ip} & e_{iv} \end{array}\right]}^{T}$ will converge to zero asymptotically.

## Numerical Simulation Results

In order to verify the proposed DFTC scheme, two different reconfigurable manipulators with 2-DOF and 4-DOF, respectively, are employed for numerical simulation. The friction terms of configuration A and configuration B are, respectively, expressed as follows.

Configuration A:

$$\begin{array}{c} F\left(q,\dot{q}\right)=[\begin{array}{c} {\dot{q}}_{1}+10\sin\left(3q_{1}\right)+2\text{sgn}\left({\dot{q}}_{1}\right)\\ 1.2{\dot{q}}_{2}+5\sin\left(2q_{2}\right)+\text{sgn}\left({\dot{q}}_{2}\right) \end{array}]. \end{array}$$ (63)

Configuration B:

$$\begin{array}{c} F\left(q,\dot{q}\right)=[\begin{array}{c} 2{\dot{q}}_{1}+5\sin\left(2q_{1}\right)+\text{sgn}\left({\dot{q}}_{1}\right)\\ 1.5{\dot{q}}_{2}+5\sin\left(q_{2}\right)+1.2\text{sgn}\left({\dot{q}}_{2}\right)\\ 1.8{\dot{q}}_{3}+10\sin\left(2q_{3}\right)+\text{sgn}\left({\dot{q}}_{3}\right)\\ 3{\dot{q}}_{4}+2\sin\left(q_{4}\right)+2\text{sgn}\left({\dot{q}}_{4}\right) \end{array}]. \end{array}$$ (64)

The desired trajectories are given as follows.

Configuration A:

$$\begin{array}{c} q_{d}=[\begin{array}{c} q_{1d}\\ q_{2d} \end{array}]=[\begin{array}{c} \text{0.5cos(t)}+0.2\text{sin(3t)}\\ \text{0.3cos(3t)}-\text{0.5sin(2t)} \end{array}]. \end{array}$$ (65)

Configuration B:

$$\begin{array}{c} q_{d}=[\begin{array}{c} q_{1d}\\ q_{2d}\\ q_{3d}\\ q_{4d} \end{array}]=[\begin{array}{c} \text{0.1cos(4t)}+0.2\text{sin(3t)}\\ \text{0.2cos(3t)}+\text{0.3sin(2t)}\\ \text{0.3cos(2t)}+0.1\text{sin(t)}\\ \text{0.2sin(t)}+\text{0.1cos(3t)} \end{array}]. \end{array}$$ (66)

The actuator failure as the following function is injected into joint 1 of configuration A.

$$\begin{array}{c} f_{1a}=\alpha \left(t-6\right)\cdot 8. \end{array}$$ (67)

The parameters of DSMO are set as *κ*_*i*1_ = 10, *κ*_*i*2_ = 30, *σ*_*i*_ = 0.02, *η*_*ifo*_ = 0.02, *η*_*igo*_ = 0.02, *η*_*ipo*_ = 0.01 and *λ*_*i*_ = 1.

The control parameters are set as *k*_*i*1_ = 0.001, *k*_*i*2_ = 200, *ρ*_*i*1_ = 10, *ρ*_*i*2_ = 200 and *μ*_*i*_ = 0.01; the adaptive gains are *η*_*if*_ = 0.001, *η*_*ig*_ = 0.001 and *η*_*ip*_ = 500.

**Fig 2:**
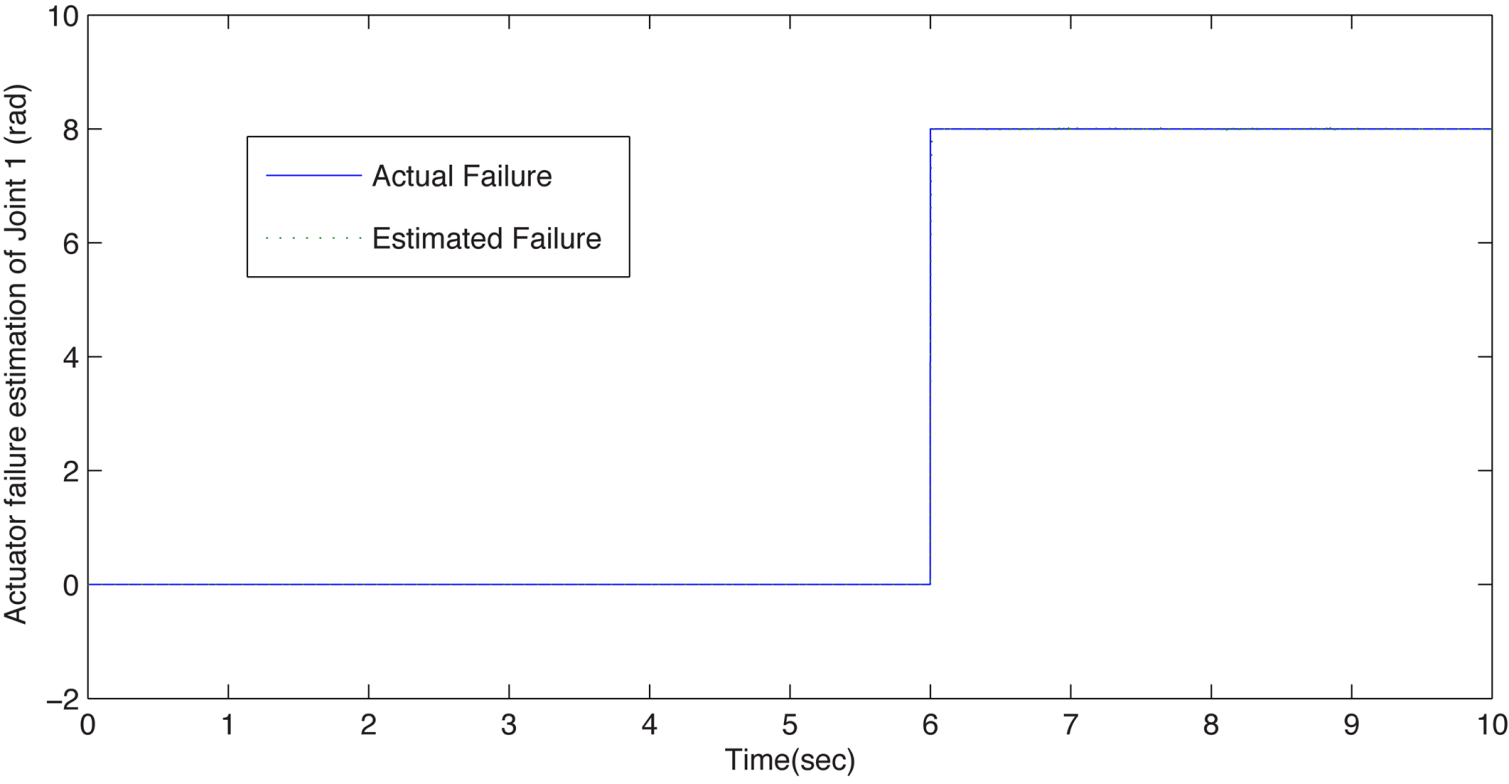
The estimated failure function curve of configuration A. The solid line and the dotted line present the actual failure and estimated failure when joint 1 suffers actuator failure at *t* = 6s, respectively. One can see that the estimated one can follow the actual one precisely in real time in the presence of actuator failure.

**Fig 3:**
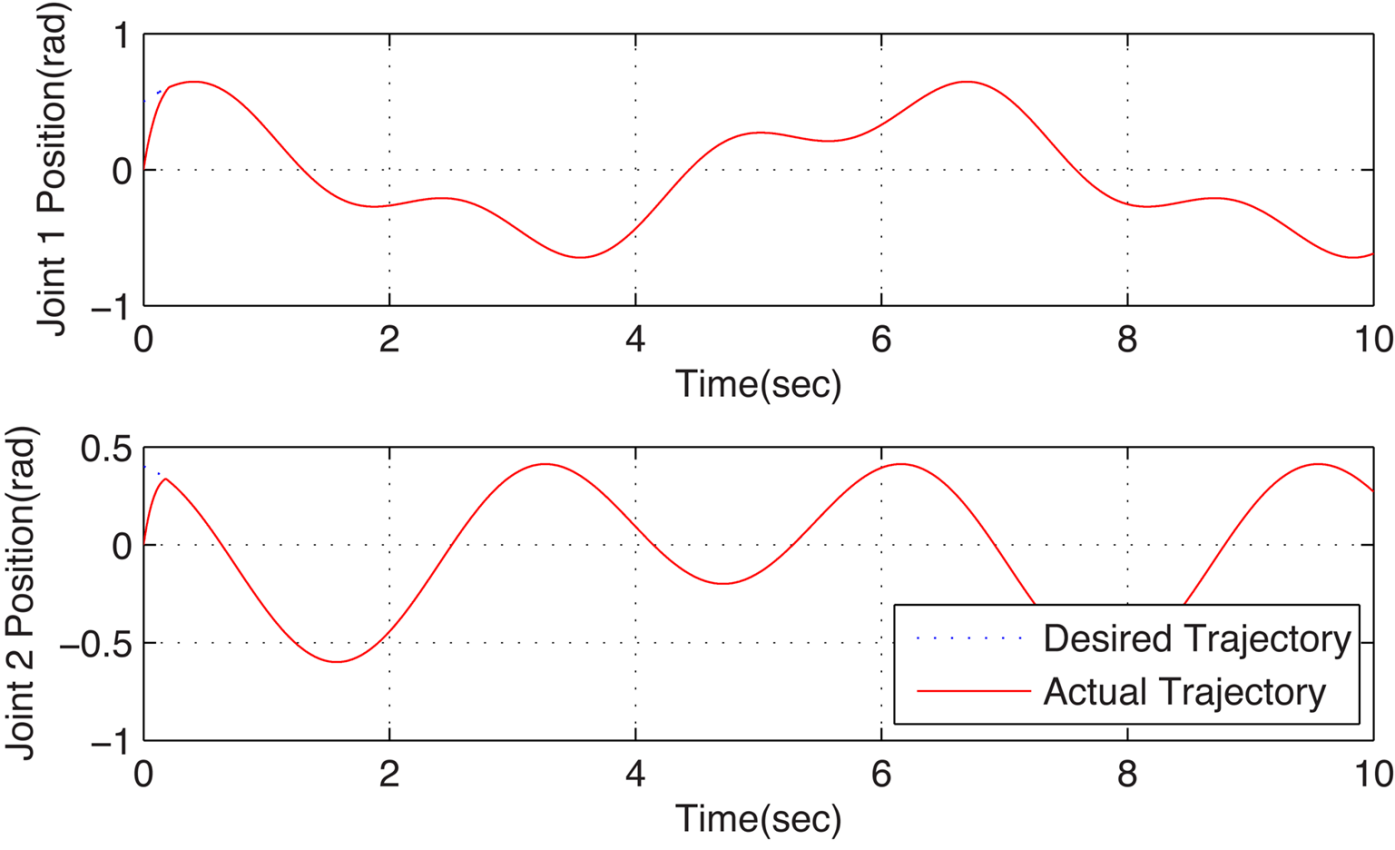
Position tracking curves of configuration A with fault tolerant control. These figures illustrate the joints position tracking performance under the proposed fault tolerant control scheme when the reconfigurable manipulator suffers actuator failure. The dotted lines and the solid lines denote the desired trajectories and the actual ones. One can see that the fault system can be recovered well.

Fig 2 illustrates the estimated fault curve and the actual one that occurred in joint 1 of configuration A. One can observe that the fault function can be estimated precisely in real-time. When the actuator failure occurs, the trajectory tracking is successfully guaranteed by the proposed dual closed-loop DFTC law, and the corresponding simulation results can be observed in Fig 3. This scheme can handle the actuator failure in the local module joint. It implies that the proposed DFTC scheme is effective to handle actuator failure in the faulty subsystem.

**Fig 4:**
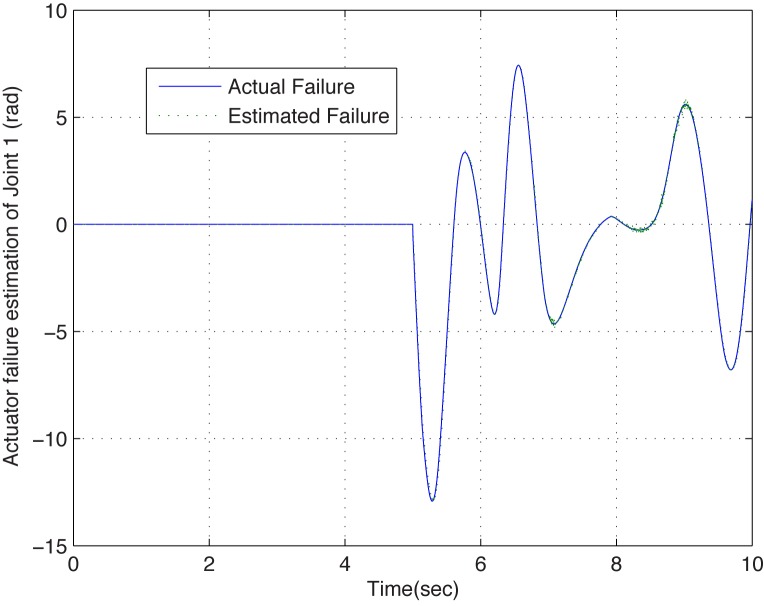
The estimated failure function curve of configuration B. The solid line and the dotted line present the actual failure and estimated failure when joint 1 suffers actuator failure at *t* = 5s, respectively. One can see that the estimated one can follow the actual one precisely in real time in the presence of actuator failure.

**Fig 5:**
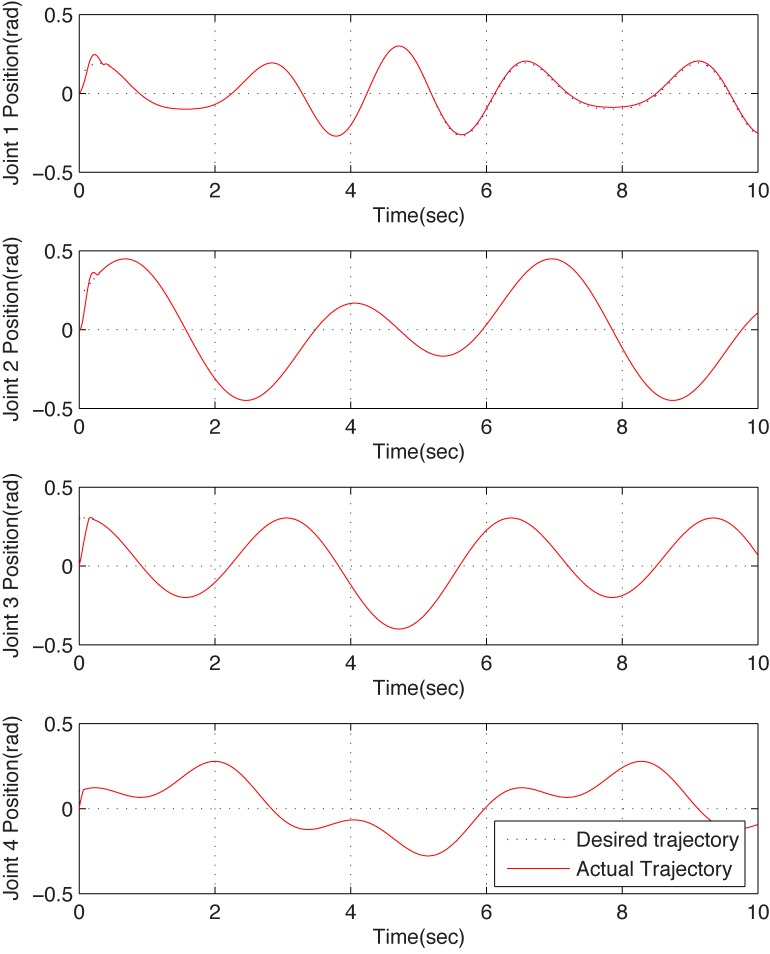
Position tracking curves of configuration B with fault tolerant control. These figures illustrate the joints position tracking performance under the proposed fault tolerant control scheme when the reconfigurable manipulator suffers actuator failure. The dotted lines and the solid lines denote the desired trajectories and the actual ones. One can see that the fault system can be recovered well.

To further test the effectiveness of the proposed scheme, a 4-DOF reconfigurable manipualtor which has a more complex structure is employed for simulation. Inject the following actuator failure function on joint 1,

$$\begin{array}{c} f_{1a}=\alpha \left(t-5\right)\cdot 10\sin\left(2q_{1}\right){\dot{q}}_{1} \end{array}$$ (68)

and the same scheme is utilized with the same control parameters. From the simulation results shown in Fig 4 and Fig 5, similar conclusions can be obtained as well. From the results, one can obtain that the failure function can be estimated in real-time precisely, regardless of what the function type is. Moreover, the dual closed-loop DFTC law, which is realized by compensating the failure estimated via DSMO, is effective.

## Discussion and Conclusion

With respect to its main property of modularization, a DFTC scheme is investigated in this paper for reconfigurable manipulator which suffers from actuator failure. The considered actuator failure function is estimated in real-time by virtue of DSMO, and then the estimated fault function is utilized to compensate the fault in the presented dual closed-loop integral sliding mode controller. Indeed, the control architecture is reconfigured for the aim to tolerate the actuator failure. Finally, the effectiveness of the proposed DFTC is demonstrated by two reconfigurable manipulators with different DOFs and configurations with the same control parameters.
